# Physical determinants of daily physical activity in older men and women

**DOI:** 10.1371/journal.pone.0314456

**Published:** 2025-02-03

**Authors:** Laura Karavirta, Timo Aittokoski, Katja Pynnönen, Timo Rantalainen, Kate Westgate, Tomas Gonzales, Lotta Palmberg, Joona Neuvonen, Jukka A. Lipponen, Katri Turunen, Riku Nikander, Erja Portegijs, Taina Rantanen, Søren Brage

**Affiliations:** 1 Faculty of Sport and Health Sciences and Gerontology Research Center, University of Jyväskylä, Jyväskylä, Finland; 2 MRC Epidemiology Unit, University of Cambridge, Cambridge, United Kingdom; 3 Department of Technical Physics, University of Eastern Finland, Kuopio, Finland; 4 Department of Emergency Care, Kuopio University Hospital, Kuopio, Finland; 5 Seinäjoki University of Applied Sciences, Seinäjoki, Finland; 6 Wellbeing Region of Central Finland/Wellbeing Services County of Central Finland, Finland; 7 University Medical Center Groningen, Center for Human Movement Sciences, University of Groningen, Groningen, the Netherlands; University of Bourgogne France Comté, FRANCE

## Abstract

**Introduction:**

The ability to perform bodily movement varies in ageing men and women. We investigated whether physical fitness may explain sex differences in daily physical activity energy expenditure (PAEE) among older people.

**Methods:**

In this cross-sectional study, a population-based cohort of 75, 80, and 85-year-old men and women (n = 409, 62% women) underwent laboratory-based assessment of walking speed, maximal knee extension strength, and body fat percentage. Free-living physical activity was assessed as total PAEE, and light (LPA) and moderate-to-vigorous physical activity (MVPA) using individually calibrated combined accelerometry and heart rate sensing. Path modelling was used to examine indirect associations between sex, physical fitness, and physical activity.

**Results:**

Men had better physical fitness and higher overall PAEE (mean 34.0 (SD 10.8) kJ/kg/day) than women (28.3 (8.4) kJ/kg/day, p<0.001). The path model for PAEE explained 33% of the variance. The direct association between sex and PAEE was non-significant, whereas the association between sex and PAEE mediated by body fat (β = 0.20, p<0.001) and walking speed (β = 0.05, p = 0.001) were statistically significant. Similarly, associations between sex and MVPA mediated by body fat (β = 0.11, p = 0.002) and walking speed (β = 0.05, p = 0.001) were significant, as were the associations between sex and LPA mediated by body fat (β = 0.24, p<0.001) and walking speed (β = 0.03, p = 0.019).

**Conclusion:**

Differences in physical activity between men and women may reflect underlying differences in cardiorespiratory fitness and adiposity. These results highlight the importance of maintaining physical fitness to support active living in older adults.

## Introduction

Low physical activity is of concern, especially in adults aged over 65, who are at risk for functional limitations and loss of independence [1, 2]. The overall volume of physical activity is typically expressed as daily physical activity energy expenditure (PAEE), which is accumulated through the engagement of everyday physical behaviours at different intensities. Wearable sensors, such as accelerometers and heart rate (HR) sensors, can be used to assess physical activity and to estimate PAEE [3, 4]. To convert data from wearables into PAEE estimates–often grouped into light, moderate, and vigorous physical activity–methodological studies with suitable criterion measures and population groups have been conducted, and estimates of absolute intensity and volume of activity have been validated [5, 6]. Using wearables to estimate PAEE makes it possible to examine relationships between health and the volume and intensity of activity, as recently demonstrated for mortality in the UK Biobank database [7]. These prospective studies provide evidence of the health consequences of different activity behaviour profiles but say little about the determinants of physical activity, including individual physical fitness, which declines faster in older than in younger people [8].

Physical fitness is a group of physical characteristics that are defined as the “physiologic attribute determining a person’s ability to perform muscle-powered work”, whereas physical activity is bodily movement produced by skeletal muscles that results in energy expenditure [9]. Therefore, instantaneous PAEE, or intensity, is dependent on the individual capacity to perform muscle-powered work at any given moment. Physical fitness is a multicomponent characteristic that includes body composition, cardiorespiratory fitness, and muscle strength, which are all independently associated with health [10–12]. It is well known that men generally have more muscle mass, higher muscle strength and better cardiorespiratory fitness than women of the same age. On average, cardiorespiratory fitness is approximately 1 to 2 METs higher and maximal strength 50% higher in older men than in women [8, 13, 14]. Conversely, the proportion of body fat is approximately 10% lower in older normal weight men than in women [15]. Together these components of physical fitness could translate to higher absolute physical activity intensity and volume in men. Thus, the previously observed tendency of men to favour vigorous intensity (> 6 or 7 MET) activities more than women, and women to engage in lower intensity (from 1.5 up to 3 or 4 MET) activities more often than men [16, 17], may be by constraint rather than by choice.

The aim of this study was to explore the direct and indirect associations between physical characteristics and physical activity volume and intensity in older people. More specifically, we examined how the components of physical fitness (body fat percentage, walking speed, and muscle strength) may mediate the relationships between sex and physical activity, enabling unbiased comparisons of physical behaviour between women and men. We hypothesized that some of the variance in physical activity would be explained by physical fitness rather than sex.

## Methods

### Participants

The data for the present analyses are from a population-based observational study: Active aging–resilience and external support as modifiers of the disablement outcome (AGNES). The study included three age cohorts: 75, 80, and 85 years old, and we have published the study protocol previously [18]. Personal details such as sex and date of birth were available in the sample specifics drawn from the Digital and Population Services Agency (https://dvv.fi/en). We targeted everyone living independently near the city centre of Jyväskylä, in Central Finland, who were born during the years specified. The Research Ethics Committee of the Central Finland Health Care District (currently named Wellbeing Services County of Central Finland) provided an approval statement on the AGNES study protocol on the 23^rd^ of August 2017. Participants were recruited between the 9^th^ of October 2017 and the 27^th^ of November 2018 and were required to provide written informed consent.

**Fig 1:**
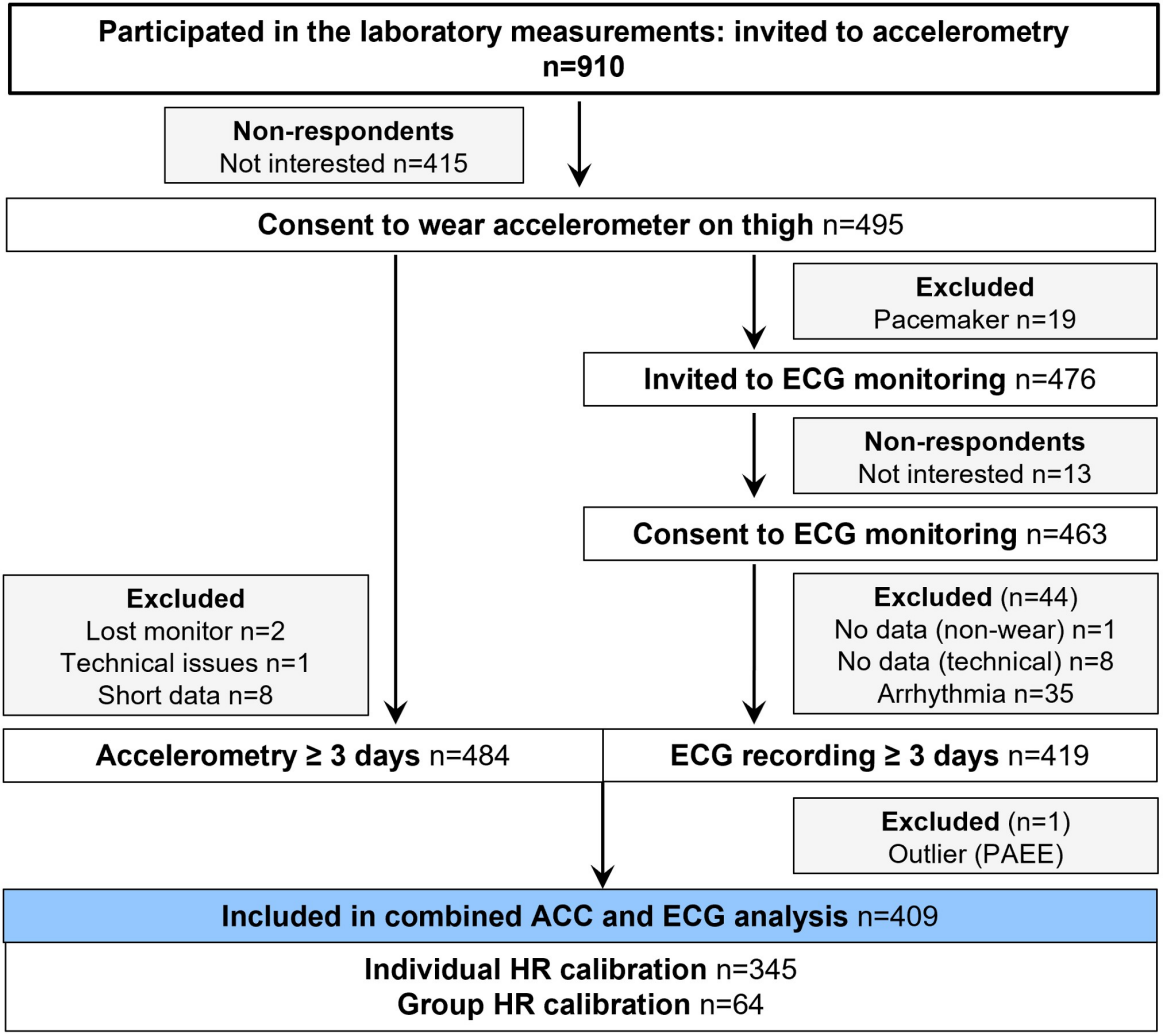
Participant flow chart. Combined wearable sensor data from the AGNES study at baseline. ACC, accelerometry; ECG, electrocardiogram; PAEE, physical activity energy expenditure; HR, heart rate.

Of the 2791 people approached, the overall participation rate was 36.6% [19]. From this sample of 1021 participants (57.3% women), 910 individuals agreed to participate in the laboratory assessments and were thus also invited to take part in device-based physical activity monitoring in free living conditions (Fig 1). To those who agreed to wear a thigh-mounted accelerometer (n = 495), we also offered an electrocardiogram (ECG) recorder unless the participant had an active implantable medical device such as a heart pacemaker (n = 19). We excluded participants with less than 3 days of wearables data, and eventually obtained combined accelerometry and HR data from 409 participants (61.9% women).

### Physical fitness measurements

#### Six-minute walk test (6MWT)

We used the 6-minute walk test to assess cardiorespiratory fitness and to individually calibrate HR to PAEE. The walking test was performed in an indoor 20-meter corridor at a self-selected usual pace [20]. Walking speed (in km/h) was calculated from the total distance walked in six minutes. The participants wore the same sensors as during the free-living monitoring: a triaxial accelerometer (sampling continuously at 100 Hz, 13-bit, ±16 g, UKK RM42; UKK Terveyspalvelut Oy, Tampere, Finland) and an ECG recorder (14-bit, ±16 g, 250 Hz, eMotion Faros 180, Bittium Corporation, Oulu, Finland). HR was derived from the ECG recordings using built-in QRS detection via Kubios HRV Premium 3.2.0 software (Kubios Oy, Kuopio, Finland), based on the Pan-Tompkins algorithm [21]. Any artifacts were corrected using automatic noise detection with manual editing, when necessary. Walking HR was calculated by averaging the resulting RR intervals (the time interval between two successive R waves on the ECG) over the last minute of the test and converting to HR in beats per minute (bpm). Recovery HR after the test was analysed from the RR intervals over 90 seconds, subjected to quadratic regression against recovery time, and solved for 45 seconds, similar to previous work [5, 22]. For PAEE estimation, HR values were expressed above sleeping HR (HRaS) but are reported as absolute values in the results for readability.

#### Maximal knee extension strength

Isometric knee extension strength was measured in a sitting position using an adjustable dynamometer chair (Metitur LTD, Jyväskylä, Finland). Maximal strength of the dominant leg was measured at an angle of 60 degrees from the fully extended leg towards flexion. Following a practice trial, the test was performed at least three times with one minute rest between trials until no further improvement occurred [18]. The highest value in newtons was recorded at the ankle, which was attached to a strain-gauge system. The result was normalized to body mass to account for the strength requirements of moving the body [23]. The test-retest reliability of the test is excellent; for measurements in 80-year-olds performed 1–2 weeks apart, the Pearson correlation coefficient was 0.965 [24].

#### Adiposity

Multi-frequency bioelectrical impedance (InBody 720, Biospace, Seoul, Korea) was used to assess fat-free mass (combining lean mass and bone mass) and adiposity (total body mass − fat-free mass) / total body mass) i.e. body fat percentage. Measurements were performed with participants wearing light clothing standing barefoot on the device platform and holding the handles in both hands. Physical assessments in the laboratory also included standard anthropometric measurements of height and body mass using a stadiometer and an electric scale (Seca, Hamburg, Germany) respectively.

### Physical activity monitoring during free-living

Physical activity monitoring took place between a home interview (where wearable sensors were attached) and a laboratory visit (where the sensors were removed). A thigh-worn triaxial accelerometer was taped to the anterior aspect of the mid-thigh of the dominant leg by a research assistant. An ECG recorder was attached with an adhesive strip that included two electrodes 12 centimetres apart. Depending on the anatomy of the participant, the strip was attached either on the sternum or diagonally on the left side of the chest under the breast to ensure comfort. Both monitors were covered with a self-adhesive film for waterproofing to enable constant wear, including during showering. However, swimming or bathing was discouraged during the monitoring period. The electrode and adhesives were replaced once in the middle of the monitoring period by a research assistant.

#### Accelerometry and ECG signal processing

Raw acceleration data were calibrated to local gravity, based on the principle described elsewhere [25]. We included only gain and offset in the optimization procedure and used the Levenberg-Marquardt algorithm to iteratively minimize the error function [20]. From the resulting gravity-calibrated acceleration values, mean amplitude deviation ($\text{MAD}=\frac{1}{n}\times \sum \left|r_{k}-r\right|$) was calculated from the vector magnitude (Euclidian norm) of the resultant acceleration ($\sqrt{X^{2}+Y^{2}+Z^{2}}$) in nonoverlapping 5-second epochs [26]. This activity-related acceleration metric is robust to residual calibration error through the subtraction operation. MAD was then resampled to 10-second epochs to match the 10-second averaging of the HR time series. The data were visually checked day-by-day to ensure that only days with complete 24-hour data without non-wear were included in the subsequent analysis. Data were excluded for 11 participants owing to either loss of monitor (n = 2), technical error (n = 1), or data availability for less than three full days (n = 8; Fig 1).

Free-living ECG recordings were analysed with commercial medically certified Awario arrhythmia analysis algorithms (Awario, Heart2Save, Kuopio, Finland) [27]. The algorithm detects and removes noisy segments of the ECG, detects the QRS complexes, and estimates the average HR in a 10-second sliding time window from the analysable parts of the ECG. Participants with persistent atrial fibrillation throughout the recording were excluded from further analyses (n = 35, Fig 1). Additionally, one outlier was excluded due to unreliable HR resulting in a PAEE estimate more than 3 SD above the sample mean.

#### Estimation of physical activity energy expenditure

PAEE was estimated from the 10-second epoch time series of MAD and HR separately, and then combined in a branched equation model [6].

*Accelerometry*. A linear regression equation for estimating instantaneous PAEE (intensity) from MAD was established using treadmill walking in a separate dataset of 12 older participants (methods described in S1 File):

$$\text{PAEE}\left(\text{J}/\text{kg}/\text{min}\right)=295.3*\text{MAD}+90.8$$ (1)

The linear equation was used for all accelerometry epochs where MAD was above a flex movement point [22]. Flex acceleration was defined as the group mean acceleration during the slowest speed of the treadmill walking (1.5 km/h), which is almost equal to the previously used flex point defined as 50% of the acceleration measured during walking at 3.2 km/h [22]. Between the flex point and zero acceleration (the lowest recorded MAD during free living), PAEE was extrapolated linearly between PAEE at the flex point and the origin of 0 g, 0 J/kg/min (S1.1 Fig in S1 File).

*Heart rate*. From the HR time-series data, PAEE was estimated using an individual calibration equation. Individual variation in the relationship between HR and energy expenditure was captured using a novel calibration method based on a self-paced walking test [5]. The method can be used to individually determine the linear relationship between HR and PAEE, which can then be used to compute PAEE at any HR level as follows:

$$PAEE_{ij}=\beta_{i}\times HRaS_{ij}+\alpha_{i}$$ (2)

where PAEE_*ij*_ and HRaS_*ij*_ are physical activity energy expenditure (J/kg/min) and heart rate (bpm above sleeping heart rate) for participant *i* for epoch *j*. *β*_*i*_ and *α*_*i*_ are the slope and intercept of the linear regression equation for participant *i*.

To calculate the individual slope and intercept of the linear equation, we computed one more parameter from the 6-minute walk test in addition to walking and recovery HR: energy pulse, which was defined as the average HR above sleeping HR divided by the energy cost of walking using a previously validated equation [28]:

$$PAEEwalk\left(Jkg^{-1}min^{-1}\right)=20.35\times \left(3.85+5.97\times walk\;speed^{2}÷height\right)$$ (3)

where 20.35 is the caloric equivalent of oxygen in J/ml O_2_ [29], walk speed is in m/s, and height is in m. Energy pulse was further transformed using a natural logarithm (ln EP). We used the combination of the simple and complex equations by Westgate and colleagues [5] with an average weighting (factor 0.5) to account for beta-blocker use and sex but to avoid parameter overfitting:

$$\beta_{i}=0.5\times \left(5.67+5.82\right)+0.5*\left(0.767+0.513\right)*\;\text{lnEP}+0.5*\left(0+0.572\right)*\text{sex}$$

$$\begin{array}{ll} \alpha_{i} & =–0.5*\left(305+311\right)+155\times \text{lnEP}-0.5*\left(1.45+1.50\right)\\ & \times \text{HRaSrec}-0.5*\left(0+0.4\right)\times \text{sex}+0.5\times \left(0+164\right)\\ & \times \text{betablocker}-0.5\times \left(0+69\right)\times \text{lnEP}\times \text{betablocker} \end{array}$$ (4)

where *i* indicates participant *i*, sex was coded 0 = woman and 1 = man, and beta-blocker use 0 = no and 1 = yes. HRaSrec is recovery heart rate 45 seconds after the walking test above sleeping HR.

The individual slope β_*i*_ and intercept α_*i*_ were used to estimate PAEE when HR was at least 5 bpm above flex HR. Flex HR is a theoretical deflection point above which HR is linearly associated with PAEE and is classically defined as “the mean of the highest HR during rest and the lowest HR during the lightest imposed exercise” (Ceesay et al., 1989). Below this point, PAEE was interpolated to 0 J/kg/min at resting HR, since at low HR levels, HR can fluctuate due to factors other than physical activity [5, 22]. Flex HR was individually estimated from the midpoint between the HR corresponding to PAEE at the slowest walking speed of our treadmill protocol (108 J/kg/min at 1.5 km/h, S1 File) and resting HR determined as 10 bpm above sleeping HR. For participants in whom we were unable to extract valid HR from the walking test (n = 64, Fig 1), we used a group calibration equation based on all participants in the study with valid calibration whilst accounting for age, sex, beta blockage, and sleeping HR:

$$\begin{array}{ll} PAEE_{j} & =(7.23-0.00878\times \text{age}+0.359*\text{sex}-0.000175\times \text{HRsleep}\\ & +0.0671\times \text{betablocker})\times HRaS_{j}-2.10\times \text{age}+18.0\\ & \times \text{sex}-0.0903\times \text{HRsleep}+59.9\times \text{betablocker}-4.27 \end{array}$$ (5)

where HRaS_*j*_ is HR in epoch *j* expressed as above sleeping HR. In the group-calibrated subsample, flex HR (above sleeping HR) was predicted from sleeping HR: 0.37 × sleeping HR − 2.5 + 5 [22]. Sleeping HR was defined as the median of the 10^th^ lowest HR observed during sleep across multiple 24-hour periods [6].

*Branched equation*. Accelerometry and HR-based PAEE time series were combined using branched equation modelling [6]. In essence, the model gives a larger weight to accelerometry-based PAEE estimation when acceleration and HR are low (due to the known fluctuation in HR at low levels regardless of physical activity), and a larger weight to HR when it is above the flex point and accompanied by physical movement, implemented as described elsewhere [3]. In addition, whenever HR was not available due to noise or transient arrhythmia in the ECG signal, acceleration-PAEE was used. HR availability is reported as a percentage of the accelerometer wear time.

In addition to overall PAEE, we calculated PAEE at moderate-to-vigorous (MVPA, 2–5 net METs) and light intensity (LPA, 0.5–2 net METs) as minutes and proportions of the overall PAEE [9]. MVPA was also expressed in MET-minutes. The distribution of PAEE was additionally calculated in fine-grained intensity bins of 0.5 to 1.0 METs each. One MET was defined according to the standard of 3.5 ml/kg/min [30] and converted to J/kg/min using 20.35 J/ml [29], resulting in a value of 71 J/min/kg.

### Covariates

Age, duration of education, chronic conditions, beta-blocker use, smoking, accelerometer wear time, and HR availability as a percentage of the accelerometer wear time were tested as potential covariates. The duration of education (years) and the total number of self-reported physician-diagnosed chronic conditions were self-reported during the home interview. The complete list of prompted conditions can be found in the protocol article [18]. Participants reported their current medication in a postal questionnaire, and beta-blocker medication was identified (yes/no) based on the ATC (Anatomical Therapeutic Chemical) code. Smoking was self-reported as never, past, or current, and dichotomously reclassified as never or past/present.

### Statistical analyses

Group values are means followed by standard deviations (SD), or the median and the first and third quartile with lower and upper adjacent values for visualisation. Differences in participant characteristics and physical activity between the sexes were tested using an independent-samples t-test and Cohen’s d. Effect sizes were interpreted as small (d = 0.2), medium (d = 0.5), or large (d ≥ 0.8) [31]. Cross-tabulation and chi-squared tests were used for categorical variables. Bivariate associations between components of physical fitness and physical activity, separated by sex, were analysed using Pearson’s correlation. A p-value <0.05 was considered statistically significant. Path modelling was conducted to examine whether the components of physical fitness (body fat, walking speed, and muscle strength) mediate the relationships between sex and physical activity. The model was structured considering the previously observed heterogeneity of physical fitness within sexes, which suggests that sex alone may not account for the individual variance in physical activity. Maximum likelihood estimation with robust standard errors (MLR) was used to obtain parameter estimates assuming missing values to be missing at random (MAR). The covariance coverage was 0.968 at minimum. In separate models, a component of physical activity (i.e. overall PAEE, MVPA, or LPA) was an outcome and sex was an explanatory variable. All three physical fitness components were added simultaneously to the models to test the potential mediator effect, and they were allowed to correlate with each other. Each component of physical fitness and physical activity was adjusted for age, duration of education, and number of chronic conditions. Additionally (or based on modification indexes), age was allowed to correlate with the number of chronic conditions and the duration of education. In the final models, only statistically significant associations were included. Chi-square (χ2), root mean squared error of approximation (RMSEA, <0.06), comparative fit index (CFI, >0.95), Tucker-Lewis Index (TLI, >0.95), and standardized root mean square residual (SRMR <0.08) are reported as indices of model fit.

As a sensitivity analysis and to enhance the applicability of the present results to studies that use an accelerometer only, path modelling was also performed for accelerometry-based PAEE without HR sensing in the same sample. Statistical significance was set at p<0.05. Analysis was conducted using IBM SPSS Statistics 28.0.1.1 and Mplus version 8.6 [32].

## Results

Of the 409 participants from whom PAEE data were obtained, data were missing from one man and two women for adiposity (due to device malfunction or metal implants), three men for muscle strength, and four men and five women for walking speed (due to musculoskeletal or cardiorespiratory disorders). Men and women in the present sample did not significantly differ in terms of age, the number of chronic conditions, duration of education, or beta-blocker use (Table 1). The most prevalent conditions were musculoskeletal disease (52.8%) and vascular disease (50.6%). Men were taller, heavier and had lower body fat percentage than women. Muscle strength and walking speed were also higher in men. Women had higher HR during sleep, self-paced walking, and recovery after the walk than men.

**Table 1:** AGNES study participant characteristics.

	Women		Men		Total		
	(n = 253)		(n = 156)		(n = 409)		
	Mean	SD	Mean	SD	Mean	SD	p (sex)
**Age**	78.2	3.4	78.2	3.2	78.2	3.3	0.985
**Body height (m)**	1.58	0.05	1.72	0.06	1.64	0.09	<0.001
**Body mass (kg)**	70.1	11.8	79.1	11.3	73.5	12.4	<0.001
**Fat Free Mass (kg)**	42.1	4.5	57.2	6.9	47.9	9.2	<0.001
**Body Fat (%)**	39.0	7.1	27.0	6.7	34.5	9.1	<0.001
**Chronic conditions (count)**	3.0	1.8	2.7	1.8	2.9	1.8	0.17
**Education (years)**	11.6	4.0	12.1	4.4	11.8	4.2	0.31
**Muscle strength (N/kg)**	4.23	1.23	5.70	1.45	4.78	1.50	<0.001
**Walk Speed, 6MWT (km/h)**	4.13	0.79	4.45	0.76	4.25	0.79	<0.001
**Walk EE estim. (net stdMET)**	2.57	0.52	2.65	0.50	2.60	0.51	0.10
**HR walk (bpm)**	104.9	15.3	98.6	16.0	102.5	15.8	<0.001
**HR rec (bpm)**	88.8	15.0	84.4	14.6	87.1	15.0	0.007
**HR sleep (bpm)**	52.5	6.7	48.9	6.5	51.2	6.8	<0.001
	n	(%)	n	(%)	n	(%)	
**Past or present smoking (yes)**	35	13.8	67	43.2	102	25.0	<0.001
**Betablocker use (yes)**	91	36.0	45	28.8	136	33.3	0.138

Muscle strength, maximal isometric knee extension strength in newtons per body mass; 6MWT, six-minute walking test; EE estim., estimated energy expenditure; HR, heart rate; rec; recovery

**Fig 2:**
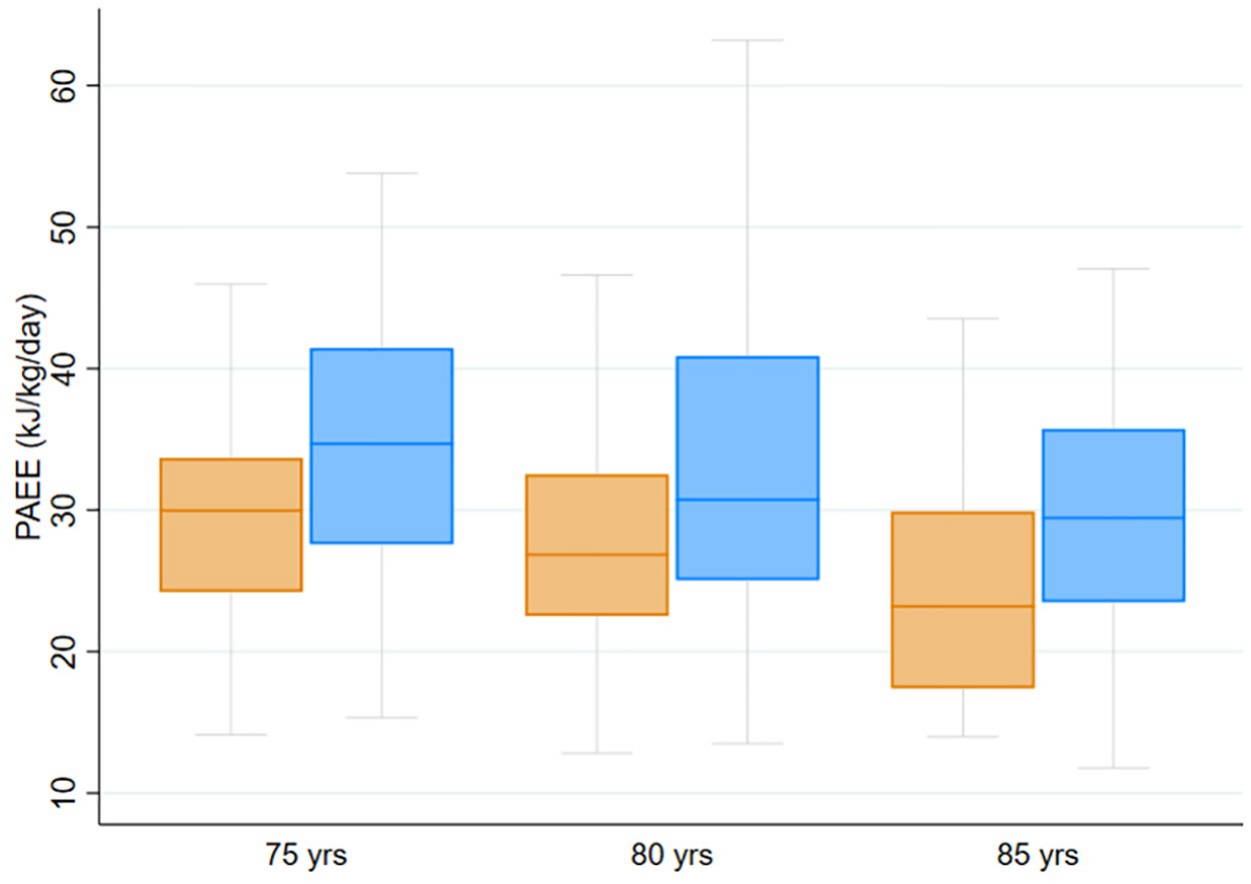
Daily physical activity energy expenditure (PAEE). A boxplot of the median (±first and third quartile with lower and upper adjacent values) unadjusted PAEE separated by age group and sex (women in orange, men in blue). PAEE was significantly higher in men and younger cohorts than in women and the oldest cohort (p<0.001).

**Fig 3:**
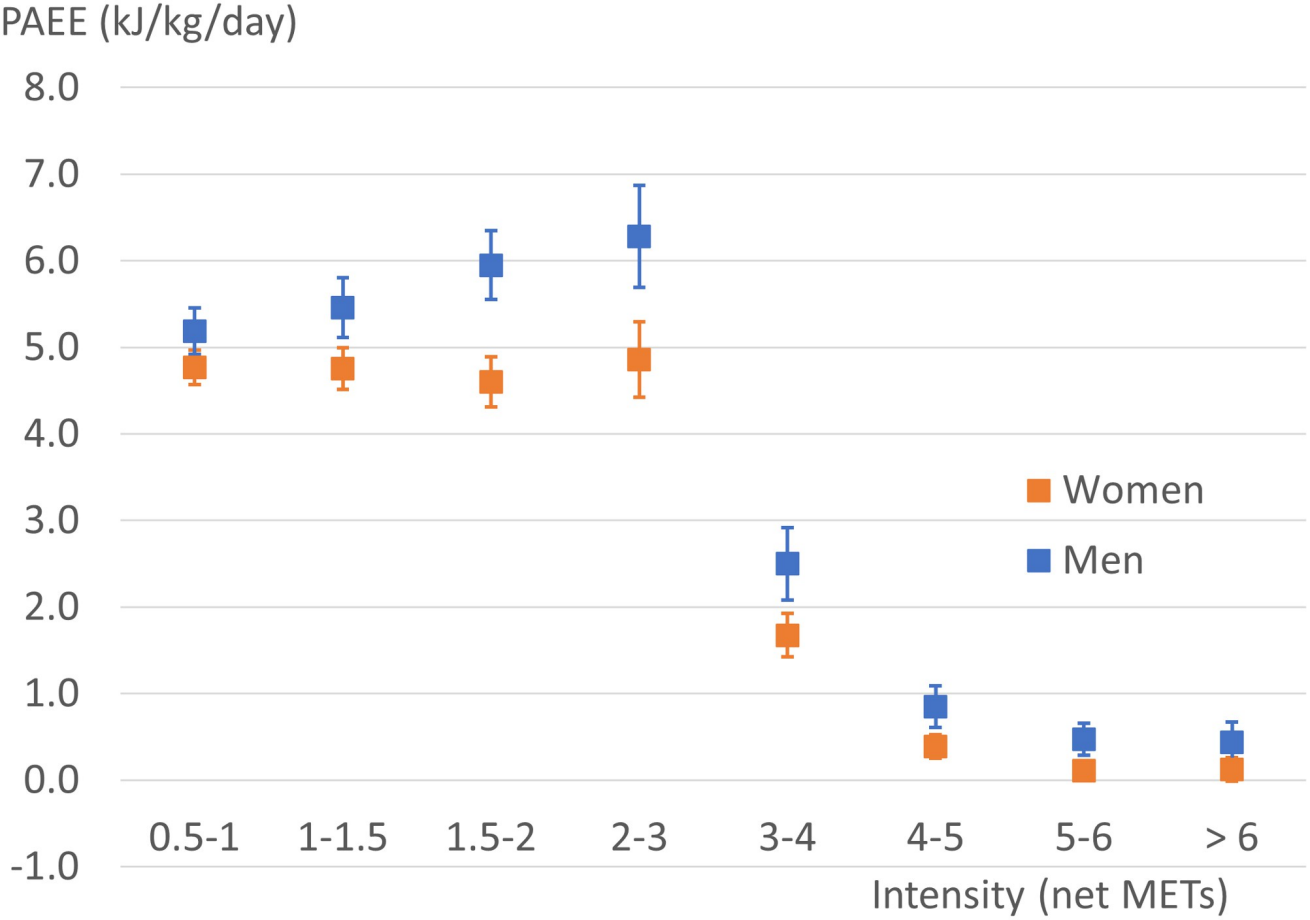
Daily accumulation of physical activity energy expenditure (PAEE) at different intensity levels. Intensity is expressed as standard metabolic equivalents above the resting level (net METs). PAEE is mean ± 95% CI in men and women. Note, bin size differences below and above 2 net METs.

Fig 2 presents overall PAEE separated by age and sex, showing inverse associations with age in both sexes. The allocation of PAEE to LPA and MVPA as energy expenditure, proportion, and minutes per day are presented in Table 2. Approximately half of the daily PAEE was spent in light-intensity activities in both men and women (p = 0.44), while the contribution of activities of at least moderate intensity was 28% in men and 23% in women (p<0.001). All the other indicators of daily physical activity were also higher in men than women (p<0.001). The effect size of sex on PAEE varied across the intensity spectrum ranging from small to medium (from 0.25 to 0.56), being largest at 1.5–2.0 METs (Fig 3). PAEE, MVPA, and LPA were significantly associated with body fat percentage, walking speed, and muscular strength in both men and women (Table 3).

**Table 2:** Physical activity metrics for men and women.

	Women		Men		Total		
	Mean	SD	Mean	SD	Mean	SD	p (sex)
**PAEE (kJ/kg/day)**	28.3	8.4	34.0	10.8	30.5	9.8	<0.001
**PAEE from MVPA (kJ/kg/day)**	7.1	5.7	10.5	7.5	8.4	6.6	<0.001
**PAEE from MVPA (%)**	22.7	12.5	28.0	13.1	24.7	13.0	<0.001
**PAEE from LPA (kJ/kg/day)**	14.1	4.8	16.6	5.4	15.1	5.2	<0.001
**PAEE from LPA (%)**	50.0	8.7	49.3	8.5	49.7	8.6	0.44
**LPA (min/day)**	184	59	211	66	194	63	<0.001
**MVPA (min/day)**	37	27	52	32	43	30	<0.001
**MVPA (min/week)**	260	187	364	227	299	209	<0.001
**MVPA (net stdMET mins/week)**	703	556	1035	741	829	652	<0.001
**Wear time (days)**	7.2	1.1	7.1	1.2	7.1	1.1	0.56
**HR availability (%)**	74.8	24.4	74.1	23.8	74.5	24.1	0.78

PAEE, physical activity energy expenditure; MVPA, moderate-to-vigorous physical activity; LPA, light physical activity; stdMET, standard metabolic equivalent (3.5 ml/kg/min)

**Table 3:** Pearson’s correlation coefficients between physical fitness and activity for men and women.

*Women*		PAEE (kJ/kg/day)	PAEE from MVPA (kJ/kg/day)	PAEE from LPA (kJ/kg/day)
Body Fat (%)	r	**-0.429**	**-0.323**	**-0.381**
	p	<0.001	<0.001	<0.001
Walk speed (km/h)	r	**0.445**	**0.425**	**0.282**
	p	<0.001	<0.001	<0.001
Muscle strength (N/kg)	r	**0.401**	**0.401**	**0.224**
	p	<0.001	<0.001	<0.001
***Men***				
Body Fat (%)	r	**-0.414**	**-0.341**	**-0.371**
	p	<0.001	<0.001	<0.001
Walk speed (km/h)	r	**0.454**	**0.440**	**0.302**
	p	<0.001	<0.001	<0.001
Muscle strength (N/kg)	r	**0.275**	**0.253**	**0.187**
	p	0.001	0.002	0.021

**Fig 4:**
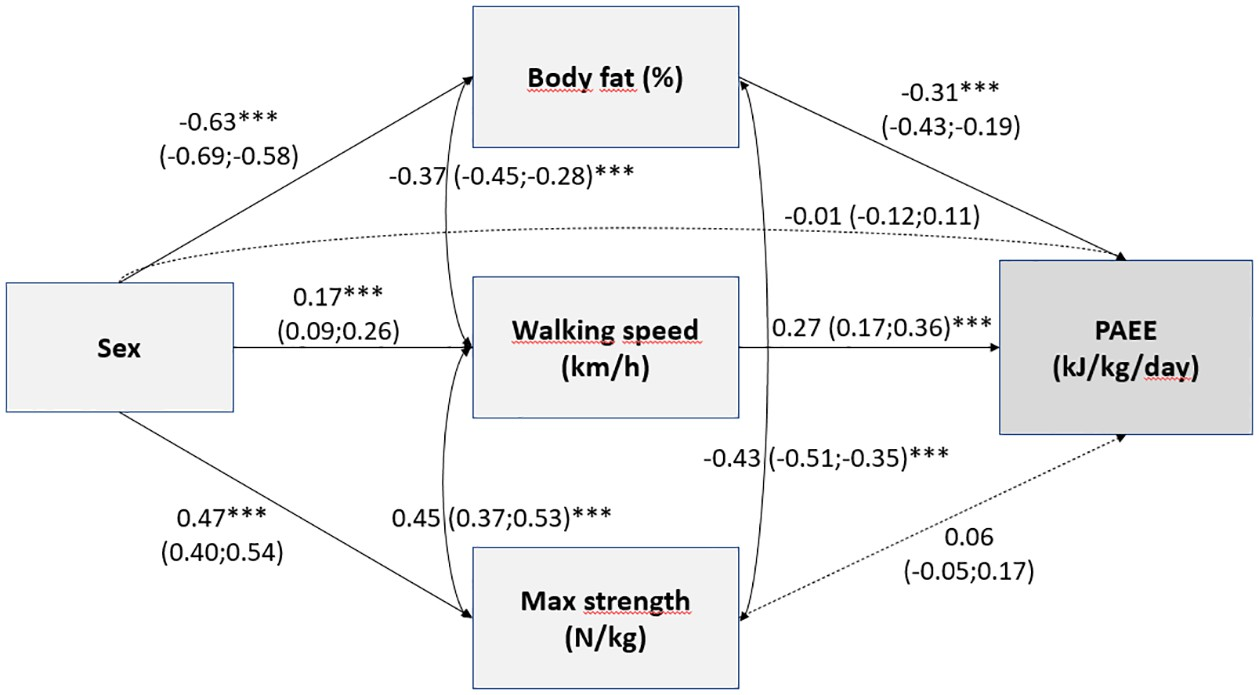
Associations of the path model for PAEE. Standardized beta coefficients (with 95% confidence intervals) from path model testing factors describing physical fitness (body fat percentage, walking speed, and muscle strength) as mediators in the relationships between sex and physical activity energy expenditure (PAEE, *p≤0.05, **p≤0.01, ***p≤0.001).

Sex had a bivariate association with overall PAEE (β = 0.28; 95% CI 0.19–0.37; p<0.001), LPA (0.23; 0.14–0.33; p<0.001), and MVPA (0.25; 0.15–0.34; p<0.001). Results of the path model further indicated that men had a lower percentage of body fat than women but faster walking speed and higher maximal strength (Fig 4). All associations of the path model are presented in S2.1 Table in S2 File. Of the physical fitness components, higher body fat percentage was negatively and higher walking speed positively associated with PAEE and MVPA. Associations between maximal strength and PAEE at different intensities were not significant. The direct effect of sex on PAEE at different intensities attenuated to non-significance.

The indirect effects of sex on PAEE mediated by body fat (β = 0.20, p<0.001) and walking speed (β = 0.05, p = 0.001) were statistically significant. The model explained 33.3% of the variance in PAEE, and the model fit was good (χ2(9) = 17.147, p = 0.047, RMSEA 0.047, CFI 0.989, TLI 0.974, SRMR 0.047). Similarly, the indirect effects of sex on MVPA (S3.1 Fig in S3 File; S2.1 Table in S2 File) and LPA (S3.2 Fig in S3 File; S2.1 Table in S2 File) mediated by body fat and walking speed were statistically significant. The models explained 27.2% of the variance in MVPA and 21.1% of the variance in LPA. Considering covariates, the number of chronic conditions was negatively associated with all physical activity variables, walking speed, and muscle strength but positively associated with body fat. Duration of education was positively associated with walking speed and negatively with age, and the number of chronic conditions was positively associated with age.

In the sensitivity analysis where only accelerometry was used (S2.2 Table in S2 File; S3.3 Fig in S3 File), all indirect effects of sex on accelerometry-based PAEE mediated by physical fitness components were significant: body fat (β = 0.16, p<0.001), walking speed (β = 0.05, p = 0.002), and muscle strength (β = 0.05, p = 0.042), collectively explaining 30.6% of the variance in accelerometry-based PAEE (χ2(9) = 16.075, p = .065, RMSEA 0.044, CFI 0.990, TLI 0.976, SRMR 0.045).

## Discussion

In this study, we used combined accelerometry and heart rate sensing to investigate the physical fitness determinants of PAEE, i.e. walking speed, adiposity, and muscular strength, in older Finnish men and women. The PAEE component from accelerometry was based on incremental walking data from a similar age group, and the PAEE component from heart rate was individually calibrated using a novel method based on self-paced submaximal walking. This is the first time the self-paced walking calibration has been applied to older people, for whom it is a feasible method due to their heterogeneous functional ability. We observed approximately 20% higher PAEE in older men than in women. Our path analysis suggested that this difference was largely explained by the lower body fat percentage and higher walking speed of men. Body fat percentage was an important contributor to LPA, whereas walking speed was the most important fitness component of MVPA. The results illustrate the intertwined association between physical fitness and physical activity in the context of ageing.

Previous studies have reported similar differences between younger men and women. In the Fenland study, which included participants aged 29–64, higher overall PAEE was observed in men (59 (SD 23) kJ/kg/day) than in women (50 (20) kJ/kg/day) [17]. Schrack et al. quantified physical activity both in absolute and relative terms. Based on absolute activity counts women appeared less active. In relative terms (percentage of HR reserve), women were less sedentary and engaged in more light and moderate activity than men [33]. The authors also observed that walking speed over 400 m was lower in those who exhibited the lowest agreement between absolute and relative physical activity. This may indicate that slow walking speed is a determinant of lower absolute volume of activity. Thus, the chosen methodology affects the magnitude of observed differences between sexes, although the methods used to quantify relative and absolute intensity may also play a role [33].

The majority of physical activity of older people is composed of walking, either incidental or planned. Therefore, the intensity at which people walk in their daily lives may have a major impact on the accumulation of MVPA. It can be inferred from the present and previous studies that the preferred intensity of walking may be on either side of the common MVPA cut point of 3 METs (i.e. 2 net METs) [34, 35]. In the present sample of older adults, the average walking intensity in the laboratory test was 2.6 (SD 0.5) net METs, varying between 1.2 and 4.2 METs. Comparable walking intensity has also been reported previously when oxygen uptake was measured at “usual comfortable pace” in a diverse group aged 30–100. They walked on average at an intensity of 13.0 (SD 2.8) ml/kg/min [35], which is equivalent to an energy cost of 2.7 standard net METs. Although preferred speed may be higher in the laboratory than in a free-living environment [36], those with lower walking speed in the laboratory are probably less likely to exceed the moderate intensity cut point during free-living walking. Slowing down the walking speed is the most common modification that older adults adopt to maintain outdoor mobility [37]. Although the duration of activity may be maintained, decreased walking intensity is less likely to exceed the absolute intensity cut point of MVPA. For LPA, an even more important contributor was body fat percentage. This may be explained by the differential metabolic contributions of muscle and fat tissue to energy expenditure during physical activity [38, 39]. PAEE is generally expressed relative to total body mass, as was the case in this study, which means that individuals with higher body fat percentage have less muscle mass for generating bodily movement, and more fat mass to carry during weight-bearing activities. The contribution of LPA was approximately half of the total PAEE, which may explain the importance of body composition for LPA.

We used combined sensing of accelerometry and HR for PAEE assessment to take advantage of both methods: 1) the well-established linear relationship between HR and energy expenditure across activity modes at moderate-to-vigorous intensities [4], and 2) the capability of thigh-mounted accelerometry to separate movement from non-movement at low intensities [26], where the correlation between HR and PAEE is low [4]. It has previously been shown that locomotion-based accelerometry models may underestimate PAEE in free-living conditions. In comparison, individually calibrated HR models generally agree more strongly with doubly-labelled water measures of PAEE, although individual variance is high. Combined estimates seem to minimise bias and error variation [3]. That said, correlations between combined sensing estimates and accelerometry-only estimates are relatively high, and our sensitivity analysis showed that the present findings are also applicable to accelerometry methodology. Physical fitness as a potential determinant of PAEE is a relevant consideration to all studies that use device-based assessment of volume or the intensity distribution of physical activity. Therefore, we encourage the inclusion of both fitness and physical activity assessments in epidemiological studies.

The strengths of the current study include the population-based sample of older men and women, which was reasonably balanced in terms of participant sex. Our sample included 61.9% of women, which is comparable to the national proportion of women in this age group (61.3%) in 2017 [40]. Our study also has limitations. Based on self-report, the subsample was somewhat more physically active than those who did not volunteer for the device-based monitoring. The subsample also had a faster walking speed [19]. Therefore, we cannot generalise our findings to represent the whole population of older people, which is an important but not unusual limitation when examining this age group. It is also important to note that we used indirect measurements of 6MWT and bioelectrical impedance as surrogates of cardiorespiratory fitness and adiposity, respectively.

To conclude, our findings are in line with previous studies as we observed higher volume and intensity of physical activity in a population-based sample of older men compared to women. This study adds to the current knowledge base by showing that the difference between men and women was largely determined by sex differences in adiposity and cardiorespiratory fitness. The mediation effect of muscle strength was weaker and only significant when PAEE was assessed using accelerometry alone. Our findings stress the importance of keeping fit and maintaining a healthy weight in order to support active living in older adults. Future studies might include a fitness assessment and measures of physical activity, as together they allow more unbiased comparisons between men and women.

## Supporting information

S1 File Linear conversion of acceleration to physical activity energy expenditure. (PDF)

S2 File Includes S2.1 and S2.2 Tables. (PDF)

S3 File Includes S3.1-S3.3 Figs. (PDF)
