# Supplementary material for: Physical determinants of daily physical activity in older men and women
Source: PLoS One. 2025 Feb 3;20(2):e0314456. doi: 10.1371/journal.pone.0314456 (PMC11790164; doi:10.1371/journal.pone.0314456)
Supplement: S2 File — (PDF) [file pone.0314456.s002.pdf]

Table S2.1. Results of path modelling testing components of physical fitness as mediators in the relationships between sex and daily physical activity energy expenditure (PAEE), moderate-to-vigorous physical activity (MVPA), and light physical activity (LPA) (n=409).

	**Associations between the model variables**					**Indirect effects**				
	**B**	**SE**	**β**	**95 % CI**	**p**	**B**	**SE**	**β**	**95 % CI**	**p**
**Outcome: PAEE**										
Sex→PAEE	-0.13	1.13	-0.01	-0.12; 0.11	.911					
Sex→Body fat (%)	-11.75	0.69	-0.63	-0.69; -0.58	<.001					
Body fat (%)→PAEE	-0.33	0.07	-0.31	-0.43; -0.19	<.001	3.91	0.82	0.20	0.12;0.27	<.001
Sex→Walk speed	0.28	0.07	0.17	0.09; 0.26	<.001					
Walk speed→PAEE	3.29	0.63	0.27	0.17; 0.36	<.001	0.92	0.30	0.05	0.02;0.08	.001
Sex→ Muscle strength	1.44	0.13	0.47	0.40; 0.54	<.001					
Muscle strength→PAEE	0.40	0.38	0.06	-0.05; 0.17	.279	0.58	0.52	0.03	-0.02;0.08	.269
Body fat (%) with Walk speed	-1.77	0.25	-0.37	-0.45; -0.28	<.001					
Body fat (%) with Muscle strength	-3.69	0.48	-0.43	-0.51; -0.35	<.001					
Walk speed with Muscle strength	0.40	0.04	0.45	0.37; 0.53	<.001					
Chronic conditions→PAEE	-0.73	0.22	-0.14	-0.22; -0.06	.001					
Chronic conditions→Body fat (%)	0.74	0.20	0.15	0.07; 0.23	.001					
Age→Walk speed	-0.05	0.01	-0.20	-0.31; -0.10	<.001					
Chronic conditions→Walk speed	-0.13	0.02	-0.30	-0.38; -0.21	<.001					
Duration of education→Walk speed	0.02	0.01	0.09	0.02; 0.17	.010					
Age→Muscle strength	-0.07	0.02	-0.15	-0.31; -0.10	<.001					
Chronic conditions→Muscle strength	-0.13	0.04	-0.16	-0.24; -0.07	.001					
Age with Duration of education	-1.86	0.66	-0.13	-0.22; -0.04	.003					
Age with Chronic conditions	1.10	0.30	0.18	0.09; 0.27	<.001					
**Outcome: MVPA**										
Sex→MVPA	0.34	0.76	0.03	-0.09; 0.12	.656					
Sex→Body fat (%)	-11.76	0.69	-0.63	-0.69; -0.58	<.001					
Body fat (%)→MVPA	-0.13	0.04	-0.17	-0.28; -0.07	.001	1.49	0.55	0.11	0.04;0.18	.002
Sex→Walk speed	0.28	0.07	0.17	0.09; 0.26	<.001					
Walk speed→MVPA	2.36	0.43	0.28	0.19; 0.37	<.001	0.66	0.21	0.05	0.02;0.08	.001
Sex→Muscle strength	1.44	0.13	0.47	0.40; 0.54	<.001					
Muscle strength→MVPA	0.45	0.25	0.10	-0.01; 0.21	.074	0.65	0.35	0.05	-0.00;0.10	.068
Body fat (%) with Walk speed	-1.76	0.25	-0.37	-0.45; -0.28	<.001					

	Associations between the model variables					Indirect effects				
	B	SE	β	95 % CI	p	B	SE	β	95 % CI	p
**Outcome: MVPA**										
Body fat (%) with Muscle strength	-3.69	0.48	-0.43	-0.51; -0.35	<.001					
Walk speed with Muscle strength	0.40	0.04	0.45	0.37; 0.53	<.001					
Chronic conditions→MVPA	-0.46	0.14	-0.13	-0.21; -0.05	.001					
Chronic conditions→Body fat (%)	0.74	0.20	0.15	0.07; 0.23	<.001					
Age→Walk speed	-0.05	0.01	-0.20	-0.30; -0.10	<.001					
Chronic conditions→Walk speed	-0.13	0.02	-0.30	-0.38; -0.21	<.001					
Duration of education→Walk speed	0.02	0.01	0.09	0.02; 0.16	.010					
Age→Muscle strength	-0.07	0.02	-0.15	-0.23; -0.08	<.001					
Chronic conditions→Muscle strength	-0.13	0.02	-0.16	-0.24: -0.07	.001					
Age with Duration of education	-1.86	0.66	-0.13	-0.22: -0.04	.003					
Age with Chronic conditions	1.10	0.30	0.18	0.09; 0.27	<.001					
**Outcome: LPA**										
Sex→LPA	-0.34	0.66	-0.03	-0.16; 0.09	.605					
Sex→ Body fat (%)	-11.76	0.69	-0.63	-0.69; -0.57	<.001					
Body fat (%)→LPA	-0.22	0.04	-0.38	-0.52; -0.25	<.001	2.66	0.50	0.24	0.15;0.34	<.001
Sex→ Walk speed	0.28	0.07	0.17	0.09; 0.26	<.001					
Walk speed→ LPA	0.99	0.35	0.15	0.05; 0.26	.005	0.28	0.12	0.03	0.00;0.05	.019
Sex→ Muscle strength	1.44	0.13	0.47	0.40; 0.54	<.001					
Muscle strength→LPA	-0.13	0.21	-0.04	-0.16; 0.08	.555	-0.18	0.31	-0.02	-0.08;0.04	.560
Body fat (%) with Walk speed	-1.76	0.25	-0.37	-0.45; -0.28	<.001					
Body fat (%) with Muscle strength	-3.68	0.48	-0.43	-0.51; -0.35	<.001					
Walk speed with Muscle strength	0.39	0.04	0.45	0.37; 0.53	<.001					
Chronic conditions→LPA	-0.28	0.13	-0.10	-0.19; -0.01	.032					
Chronic conditions→Body fat (%)	0.74	0.20	0.15	0.07: 0.23	<.001					
Age→Walk speed	-0.05	0.01	-0.20	-0.31; -0.10	<.001					
Chronic conditions→Walk speed	-0.13	0.02	-0.30	-0.38; -0.21	<.001					
Duration of education→Walk speed	0.02	0.01	0.09	0.02; 0.16	.010					
Age→Muscle strength	-0.07	0.02	-0.16	-0.23; -0.08	<.001					
Chronic conditions→Muscle strength	-0.13	0.04	-0.15	-0.24; -0.07	.001					
Age with Duration of education	-1.86	0.66	-0.13	-0.22: -0.04	.003					
Age with Chronic conditions	1.10	0.30	0.18	0.09; 0.27	<.001					

Table S2.2. Results of sensitivity analysis of path modelling testing components of physical fitness as mediators in the relationship between sex and physical activity energy expenditure measured using accelerometry (PAEE_ACC) (n=409).

	**Associations between the model variables**					**Indirect effects**				
	**B**	**SE**	**β**	**95 % CI**	**p**	**B**	**SE**	**β**	**95 % CI**	**p**
Sex→ PAEE_ACC	-0.91	0.99	-0.06	-0.18; 0.06	.349					
Sex→ Body fat (%)	-11.77	0.69	-0.64	-0.69; -0.58	<.001					
Body fat (%)→PAEE_ACC	-0.22	0.06	-0.26	-0.38; -0.13	<.001	2.60	0.73	0.16	0.08;0.25	<.001
Sex→ Walk speed	0.28	0.07	0.17	0.09; 0.26	<.001					
Walk speed→ PAEE_ACC	2.56	0.50	0.26	0.16; 0.36	<.001	0.72	0.24	0.05	0.02;0.07	.002
Sex→ Muscle strength	1.44	0.13	0.47	0.40; 0.54	<.001					
Muscle strength→ PAEE_ACC	0.57	0.30	0.11	0.00; 0.22	.047	0.82	0.41	0.05	0.00;0.10	.042
Body fat (%) with Walk speed	-1.77	0.25	-0.37	-0.45; -0.29	<.001					
Body fat (%) with Muscle strength	-3.69	0.48	-0.43	-0.51: -0.35	<.001					
Walk speed with Muscle strength	0.39	0.04	0.45	0.37; 0.53	<.001					
Chronic conditions→PAEE_ACC	-0.65	0.19	-0.15	-0.24; -0.07	.001					
Chronic conditions→Body fat (%)	0.75	0.20	0.15	0.07; 0.23	<.001					
Age→Walk speed	-0.05	0.01	-0.20	-0.31; -0.10	<.001					
Chronic conditions→Walk speed	-0.13	0.02	-0.30	-0.38; -0.21	<.001					
Duration of education→Walk speed	0.02	0.01	0.09	0.02; 0.16	.010					
Age→Muscle strength	-0.07	0.02	-0.15	-0.23; -0.08	<.001					
Chronic conditions→Muscle strength	-0.13	0.02	-0.16	-0.24; -0.07	.001					
Age with Duration of education	-1.86	0.66	-0.13	-0.22: -0.04	.003					
Age with Chronic conditions	1.10	0.30	0.18	0.09; 0.27	<.001					
